# Supplementary material for: Isolation and Characteristics of a Novel Aichivirus D from Yak
Source: Microbiol Spectr. 2023 Apr 25;11(3):e00099-23. doi: 10.1128/spectrum.00099-23 (PMC10269754; doi:10.1128/spectrum.00099-23)
Supplement: Supplemental file 1 — Supplemental material. Download spectrum.00099-23-s0001.pdf, PDF file, 2.1 MB [file spectrum.00099-23-s0001.pdf]

Supplementary appendix

Methods details of qRT-PCR for detecting AiV-D

A SYBR Green real-time RT-PCR assay was established for detecting AiV-D. Primers for AiV-D was design using Primer Premier 5.0 software and was synthesized by the Sangon Biotech (Chengdu, China). The primer sequences was based on the conserved 3D sequences for AiV-D (5'-CGCTGTCTGGAGAACCCTGAGTA-3', AiV-D-R: 5'-GTTCGATGATACCACCAAGGAGC-3'). The primers for AiV-D was used to amplify a 157 bp region of the 3D gene. The PCR systems used were as follows: 12.5 μl of SYBR Green Premix Ex Taq II (TaKaRa); 1 μl forward primer (0.05 μM); 1 μl reverse primer (0.05 μM); 2 μl of cDNA; and 8.5 μl nuclease-free water. The conditions for the PCR were as follows: 94℃ for 1 min, followed by 40 cycles at 95℃ for 15 s, 56℃ for 30 s; with the melt curve stage of 95℃ for 15 s, 60℃ for 1 min.

The 157 bp product of the 3D gene was cloned into the pMD-19-T vector (TaKaRa). The recombinant plasmid was named AiV-D-3D-pMD-19-T and was verified by sequencing by Sangon Biotech (Chengdu, China) (Fig.1). The plasmid concentration was determined with a NanoDrop one (Thermo Scientific). The DNA copy number was calculated using the following formula: copy number (copies/μL) = $[6.02 \times 10^{14} \times$plasmid concentration (μg/mL)]/[DNA length (nucleotides) $\times$ 660], the plasmid concentration was 57.5 ng/μL, the copy number was 1.84×10^{10} copies/μL.

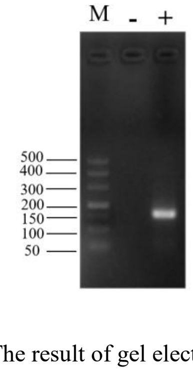

Fig.1 The result of gel electrophoresis

Note: M: DL500 DNA marker; -: Negative control; +: AiV-D positive sample

The standard curve of AiV-D was constructed using the standard plasmids with

dilutions from 10^8 to 10^2 copies/μL. The equation for the standard curve was $y = -3.55x + 41.99$ for AiV-D (Fig.2), and the R^2 value was 0.998. The specific melting peak for AiV-D (Tm = 84±0.5°C) was obtained (Fig.3).

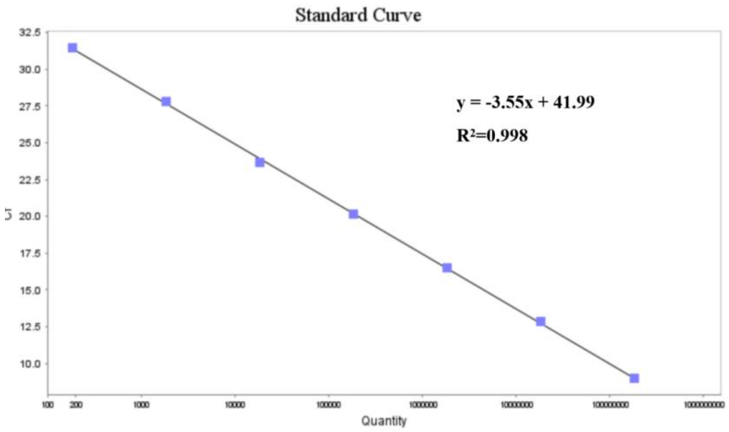

Fig.2 Standard curve of real-time RT-PCR for Aichivirus D

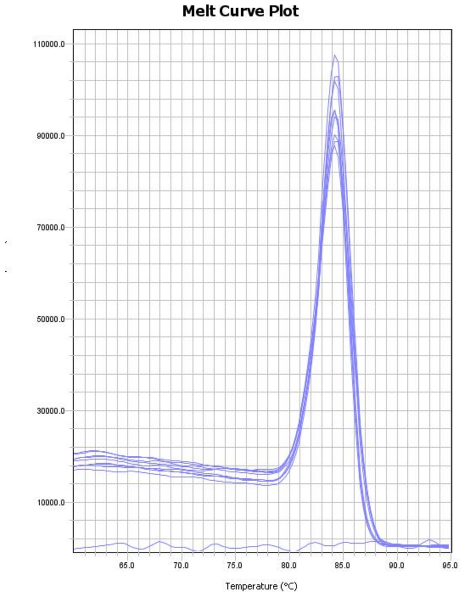

Fig.3 Melting curve of real-time RT-PCR for Aichivirus D

To test the specificity of this method, AiV-D standard plasmids was used as control samples, and extracted nucleic acid of 9 other enteric pathogens, including BRVA, BCoV, BVDV, BNeV, BNoV, BToV, Salmonella, ETEC and C.andersoni were used as templates, none of them showed a positive signal (Fig.4).

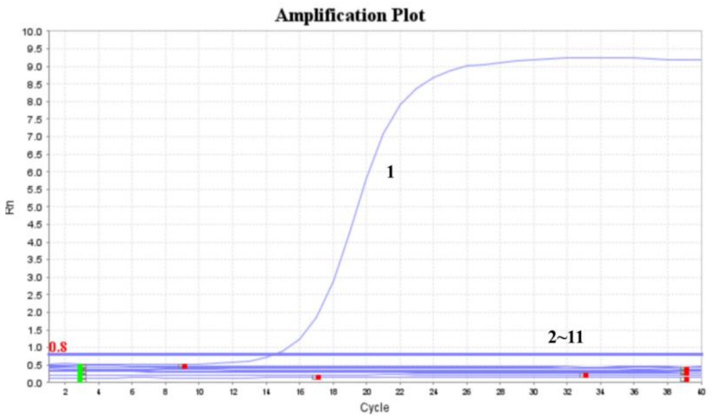

Fig.4 The specificity test of real-time RT-PCR for Aichivirus D

Note: 1: positive samples; 2~10: BRVA, BCoV, BVDV, BNeV, BNoV, BToV, Salmonella, ETEC, and C.andersoni; 11: Negative control

Standard plasmid concentrations for AiV-D (10^1~10^7 copies/μL) were applied to the optimized real-time PCR assay to determine the sensitivity of the method, the detection limits of AiV-D was 10^1 copies/μL (Fig.5).

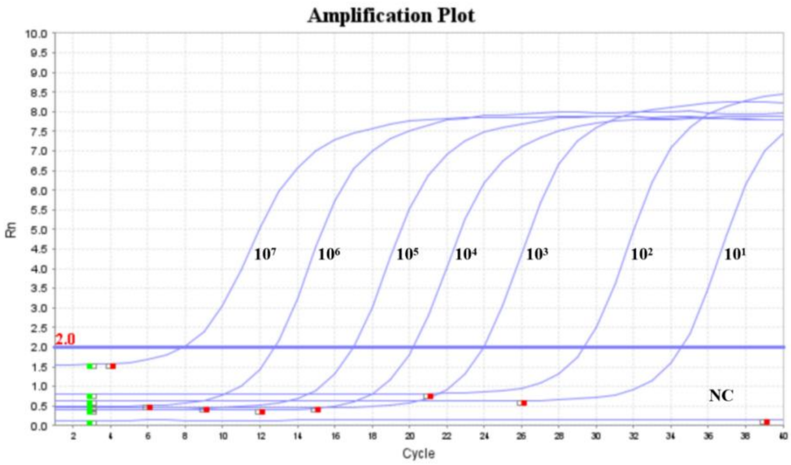

Fig.5 The sensitivity test of real-time RT-PCR for Aichivirus D

Note: 10^1~10^7: 1.84×10^1~1.84×10^7 copies/μL positive plasmid; NC: Negative control

Different concentrations (10^3~10^6 copies/μL) of the standard recombinant plasmids of AiV-D were prepared to evaluate the reproducibility of the established assay.The intra-assay variability of real-time PCR ranged from 0.90% to 1.94%, and the inter-assay variability ranged from 0.46% to 3.67%. These results showed that our

real-time PCR assay is reliable with high reproducibility for the detection of AiV-D (Table 1).

Table 1 Stability of the AiV-D real-time PCR assay method

（copies/μL）	Intra-assay variability		Inter-assay variability	
	Ct（X ± SD）	CV (%)	Ct（X ± SD）	CV (%)
1.84×10^3	28.174±0.254	0.90	29.015±0.133	0.46
1.84×10^4	24.669±0.242	0.98	24.344±0.330	1.36
1.84×10^5	19.250±0.216	1.12	20.358±0.747	3.67
1.84×10^6	16.336±0.318	1.94	16.541±0.437	2.64

In summary, this method has high specificity, sensitivity, and reproducibility, the detection limit of this assay was determined to be 18.4 copies/μL for AiV-D.

Table S1. Primers for amplifying and sequencing the genome of the AiV D

Primer	Sequence (5′→3′)	Fragment length
AichiD-1F	CTCCTCCACATCCCTTTTCG	837
AichiD-1R	AGGGAAGTGAAAAGGTCTCT	
AichiD-2F	CCGACACCATTTACACCCT	847
AichiD-2R	CCATTGCTGTGGATCTTTGA	
AichiD-3F	CACAACTACCAAAAGTGGTG	814
AichiD-3R	CACAACGGTCCAGGGACAA	
AichiD-4F	GCCCGTCAAGTTCCAGTTG	795
AichiD-4R	CCAAATTGCAAAGAAGGGAAT	
AichiD-5F	CTATTCTCCTCCTGGCACC	743
AichiD-5R	CGTTATAACAATCCGCCAACT	
AichiD-6F	TGATACTCTCCCTCTCCTCC	844
AichiD-6R	AGACAAAGGTGGTGCAGTT	
AichiD-7F	GGAACAAATTTCCCTTTCCCG	911
AichiD-7R	TGAGAGGCCTAATTTGTTGGC	
AichiD-8F	TGGCTATCTCCTTGTCATTTT	720
AichiD-8R	TCATAAAACTCGCAGTCCGC	
AichiD-9F	TCAAAAACTTCACCACTGTCC	795
AichiD-9R	GAACATGAGAAGAGGTCAGG	
AichiD-10F	AAACCTTTGATGAACTCGTG	885
AichiD-10R	TTGCCGCGTTGTAAACGTA	
AichiD-11F	CACGGGAACAACTACACGTA	791
AichiD-11R	CCTTCTTCACTTTTTCCAATGG	
AichiD-12F	GATACCCCTGGAACACCATG	758
AichiD-12R	TCAGAAGTCTTAGAGGCAGG	

Table S2. BKV/Yak/QHHN16/2021/CHN nt identity with other Aichivirus genome sequence

Gene region	Nucleotide identity (%)/amino acid identity (%)							
	Aichivirus A	Aichivirus B	Aichivirus C	Aichivirus D1	Aichivirus D2	Novel Genotype Aichivirus D	Aichivirus E	Aichivirus F
	Human/A846/88	Bovine/U-1	Caprine /F11	Bovine/Kago-1-22	Bovine/Kago-2-24	Ovine/AB18	Rabbit/01	Bat/BtMf-picov-2
5′-UTR	33.6/-	58.4/-	46.2/-	-	84.9/-	84.5/-	32.3/-	-
L	42.0/**37.4**	39.6/**34.2**	34.9/**26.6**	67.1/**61.3**	78.6/**82.0**	76.4/**77.9**	28.1/**18.9**	28.8/**18.0**
VP0	57.7/**49.9**	58.8/**57.3**	61.7/**59.1**	71.2/**76.5**	80.0/**88.0**	78.7/**84.7**	47.7/**36.8**	52.9/**42.7**
VP3	62.3/**55.5**	64.0/**64.5**	63.6/**60.4**	74.5/**80.4**	79.1/**86.9**	77.3/**83.7**	60.2/**58.0**	38.2/**22.0**
VP1	51.2/**39.1**	57.4/**49.7**	50.6/**44.6**	65.4/**70.8**	79.5/**88.5**	77.6/**84.3**	42.1/**29.2**	29.8/**19.2**
2A	48.3/**46.6**	60.2/**54.8**	56.4/**52.7**	69.2/**65.1**	89.2/**92.5**	81.3/**81.5**	50.8/**41.1**	36.0/**23.0**
2B	45.6/**38.5**	40.3/**36.5**	45.1/**39.3**	45.2/**45.1**	78.3/**84.4**	73.8/**73.8**	33.6/**26.4**	37.5/**29.0**
2C	60.1/**54.2**	59.6/**55.3**	60.2/**55.8**	65.9/**64.8**	81.0/**86.2**	79.2/**81.0**	46.5/**20.2**	37.2/**25.5**
3A	48.3/**37.1**	46.7/**41.0**	50.0/**43.8**	57.0/**50.5**	82.7/**87.6**	73.0/**76.2**	41.3/**31.4**	27.7/**23.3**
3B	50.5/**34.3**	54.4/**51.4**	46.6/**40.0**	53.4/**54.3**	82.5/**88.6**	66.0/**77.1**	38.8/**28.6**	39.8/**25.7**
3C	57.0/**50.2**	54.7/**47.8**	52.3/**45.8**	63.6/**57.1**	83.7/**88.2**	78.3/**80.3**	47.6/**31.5**	33.8/**27.5**
3D	64.5/**64.8**	64.4/**66.1**	62.0/**64.3**	73.5/**77.8**	87.0/**92.6**	80.7/**84.8**	57.4/**55.3**	57.6/**55.9**
3′-UTR	31.0/-	56.0/-	52.3/-	77.3/-	94.2/-	90.3/-	52.0/-	24.9/-
Complete	54.7/-	55.6/-	55.3/-	64.8/-	82.4/-	78.8/-	45.8/-	43.6/-
ORF	55.6/**49.4**	56.2/**53.0**	56.2/**52.5**	66.8/**66.7**	81.8/**87.0**	77.9/**80.2**	47.8/**41.2**	49.3/**42.1**

Table S3. BKV/Yak/XZNQX7/2021/CHN nt identity with other Aichivirus genome sequence

Gene region	Nucleotide identity (%)/amino acid identity (%)							
	Aichivirus A	Aichivirus B	Aichivirus C	Aichivirus D1	Aichivirus D2	Novel Genotype Aichivirus D	Aichivirus E	Aichivirus F
	Human/A846/88	Bovine/U-1	Caprine /F11	Bovine/Kago-1-22	Bovine/Kago-2-24	Ovine/AB18	Rabbit/01	Bat/BtMf-picov-2
5′-UTR	33.1/-	58.5/-	46.4/-	-	85.9/-	85.9/-	32.1/-	-
L	42.5/**36.5**	38.2/**34.7**	33.8/**27.0**	67.1/**61.3**	79.1/**82.0**	78.9/**79.3**	28.9/**18.5**	28.3/**17.6**
VP0	57.8/**50.1**	59.4/**57.0**	61.9/**60.1**	72.2/**76.7**	80.1/**89.0**	78.3/**84.7**	48.3/**36.8**	52.6/**43.2**
VP3	62.6/**55.5**	63.9/**64.1**	63.8/**60.4**	74.6/**80.8**	79.5/**86.9**	77.4/**83.7**	60.2/**58.4**	38.2/**22.0**
VP1	50.7/**38.8**	56.7/**49.4**	50.8/**44.6**	64.5/**69.6**	79.8/**87.5**	78.1/**84.6**	42.1/**29.5**	28.6/**18.9**
2A	48.3/**46.6**	60.2/**54.8**	56.6/**52.7**	69.4/**65.1**	89.7/**92.5**	80.9/**81.5**	50.8/**41.1**	36.2/**23.0**
2B	45.6/**38.5**	40.3/**36.5**	45.1/**39.3**	45.2/**45.1**	78.3/**84.4**	73.8/**73.8**	33.6/**26.4**	27.5/**29.0**
2C	59.9/**53.9**	59.4/**55.0**	59.9/**55.6**	65.8/**64.5**	80.8/**85.6**	78.9/**80.5**	46.3/**20.4**	37.0/**25.8**
3A	48.3/**37.1**	46.7/**41.0**	50.0/**43.8**	57.0/**50.5**	82.7/**87.6**	73.0/**76.2**	41.3/**31.4**	27.7/**23.3**
3B	50.5/**34.3**	54.4/**51.4**	46.6/**40.0**	53.4/**54.3**	82.5/**88.6**	66.0/**77.1**	38.8/**28.6**	39.8/**25.7**
3C	57.0/**50.2**	54.7/**47.8**	52.3/**45.8**	63.6/**57.1**	83.7/**88.2**	78.3/**80.3**	47.6/**31.5**	33.8/**27.5**
3D	64.7/**65.0**	64.5/**65.9**	62.1/**64.1**	73.2/**77.6**	86.9/**92.0**	80.6/**84.6**	57.6/**55.3**	57.6/**55.9**
3′-UTR	30.7/-	57.8/-	52.7/-	77.3/-	93.9/-	90.6/-	52.0/-	26.0/-
Complete	54.6/-	55.5/-	55.3/-	64.8/-	82.5/-	79.0/-	45.9/-	43.5/-
ORF	55.6/**49.4**	56.1/**52.8**	56.2/**52.6**	66.8/**66.5**	81.9/**86.8**	78.0/**80.3**	48.0/**41.2**	49.1/**42.1**

Table S4. Tissue distributions of Aichivirus D in experimentally infected in yak

yaks / tissues	infection group 1	infection group 2	control group 1	control group 2
	CT			
heart	-	-	-	-
liver	-	-	-	-
spleen	-	-	-	-
lung	-	-	-	-
kidney	-	-	-	-
blood	-	-	-	-
duodenum	+（15.483±0.307）	+（16.436±0.469）	-	-
jejunum	+（19.866±0.174）	+（23.869±0.251）	-	-
ileum	+（28.155±0.153）	+（27.582±0.255）	-	-
cecum	+（30.139±0.147）	+（31.307±0.409）	-	-
colon	+（31.087±0.212）	+（31.492±0.360）	-	-
rectum	+（31.255±0.434）	+（33.288±0.513）	-	-

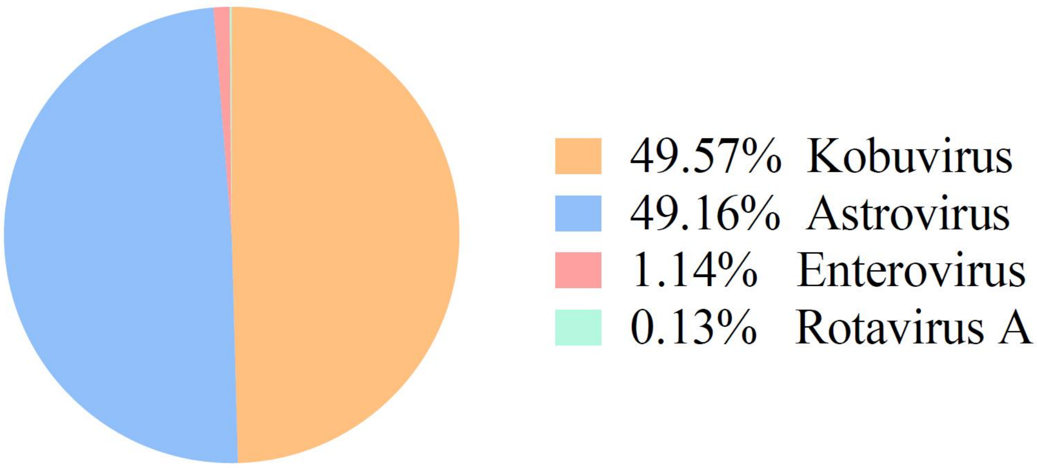

Fig S1. Sequence classifications and percentages of the viruses detected in the yak diarrheal samples.

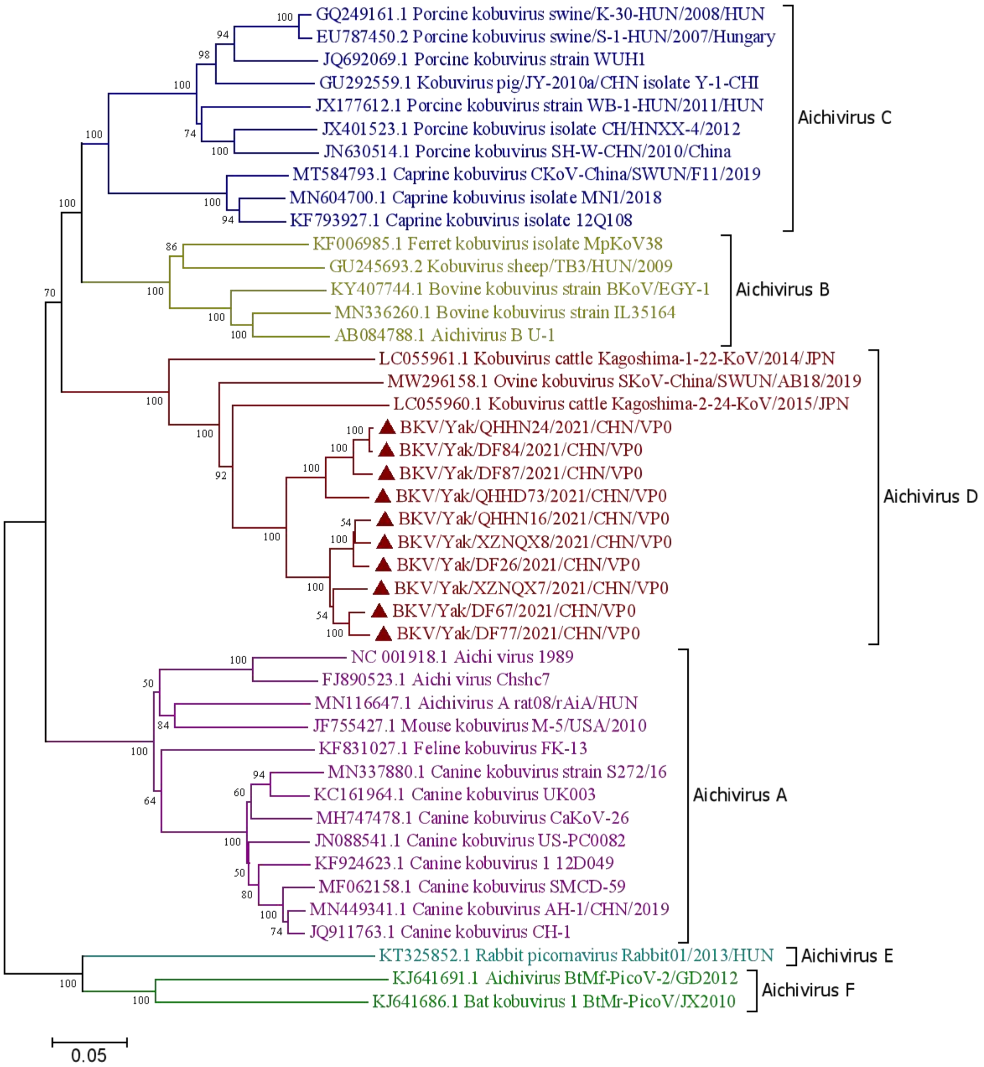

Fig S2. Phylogenetic tree of AiV VP0 gene nt sequences. Maximum-likelihood analysis in combination with 1000 bootstrap replicates was used to derive a phylogenetic tree based on the complete nucleotide sequences of AiV VP0 gene. ▲represents the AiV strains from this study.

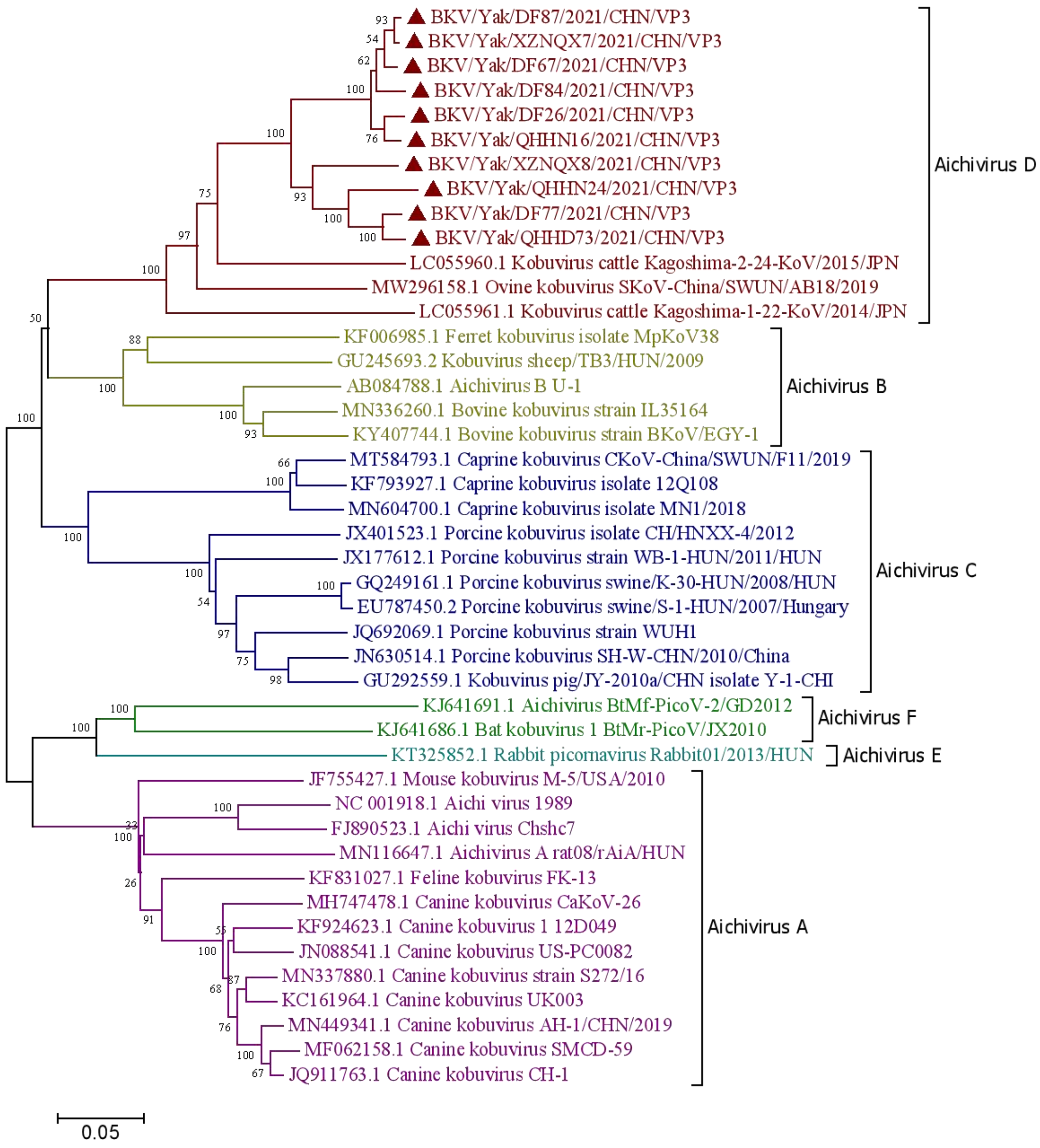

Fig S3. Phylogenetic tree of AiV VP3 gene nt sequences. Maximum-likelihood analysis in combination with 1000 bootstrap replicates was used to derive a phylogenetic tree based on the complete nucleotide sequences of AiV VP3 gene. ▲represents the AiV strains from this study.

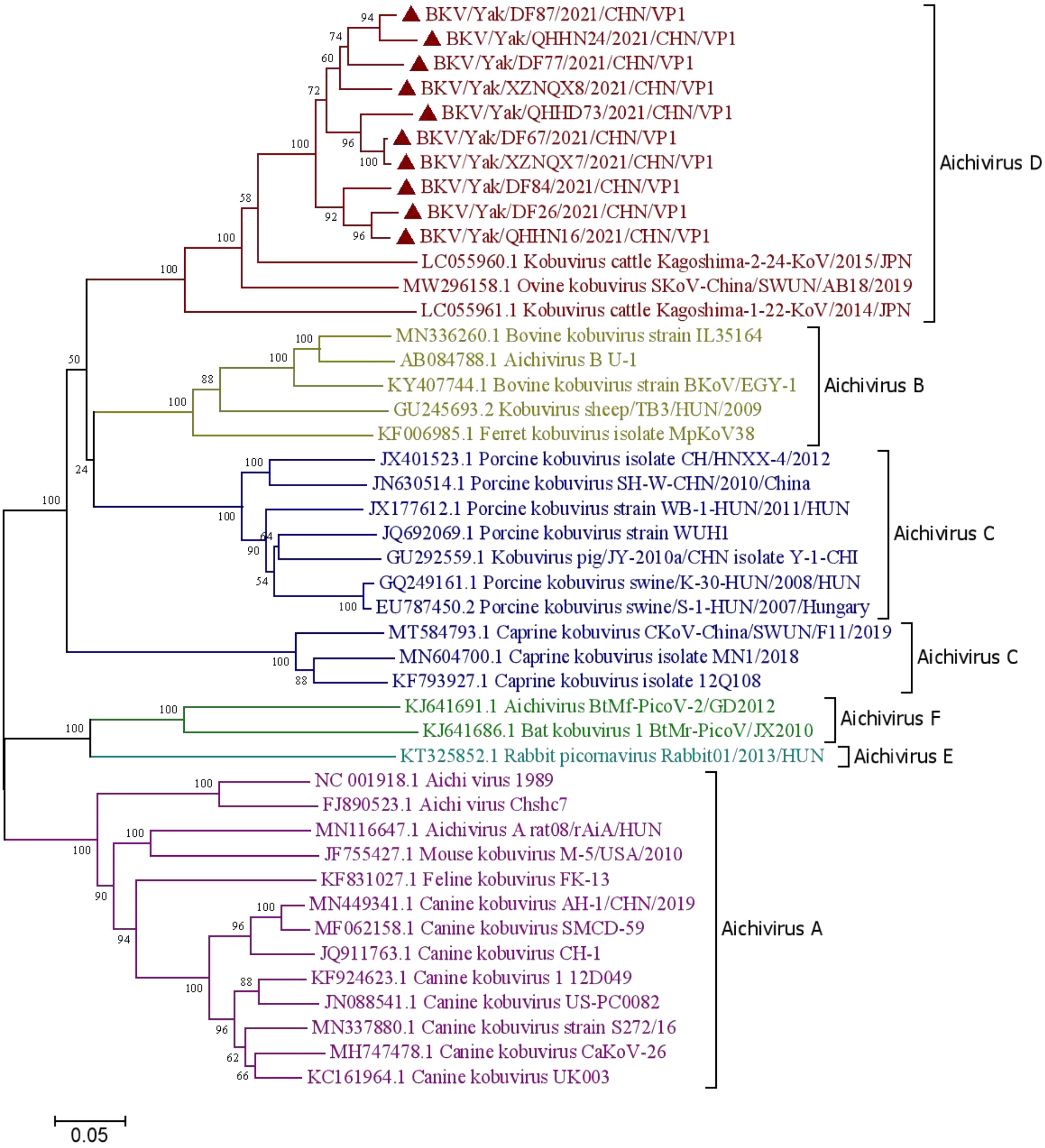

Fig S4. Phylogenetic tree of AiV VP1 gene nt sequences. Maximum-likelihood analysis in combination with 1000 bootstrap replicates was used to derive a phylogenetic tree based on the complete nucleotide sequences of AiV VP1 gene. ▲represents the AiV strains from this study.

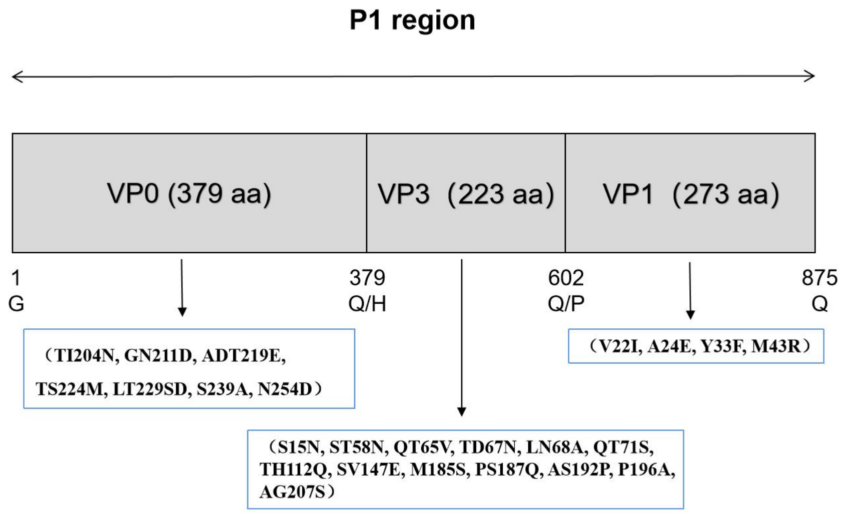

Fig S5. Schematic representation of the yak AiV D strains P1 region.

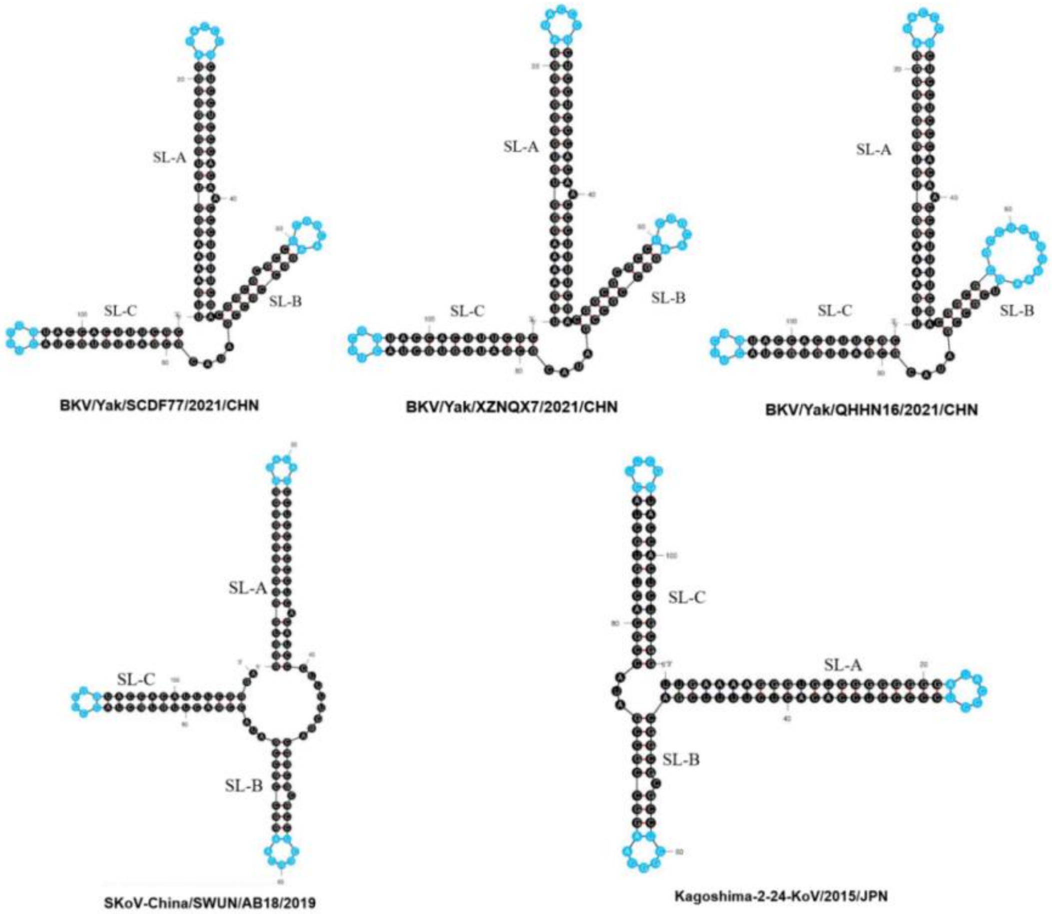

Fig S6. **The predicted secondary structures of the 5'end of the genome of AiV-D**. The positions of the 3 stem-loops are shown. Schematic of secondary structures was predicted using Mfold software (http://www.unafold.org/mfold/applications/dna-folding-form.php) and modified using RNAviz (http://rnaviz.sourceforge. Net/).

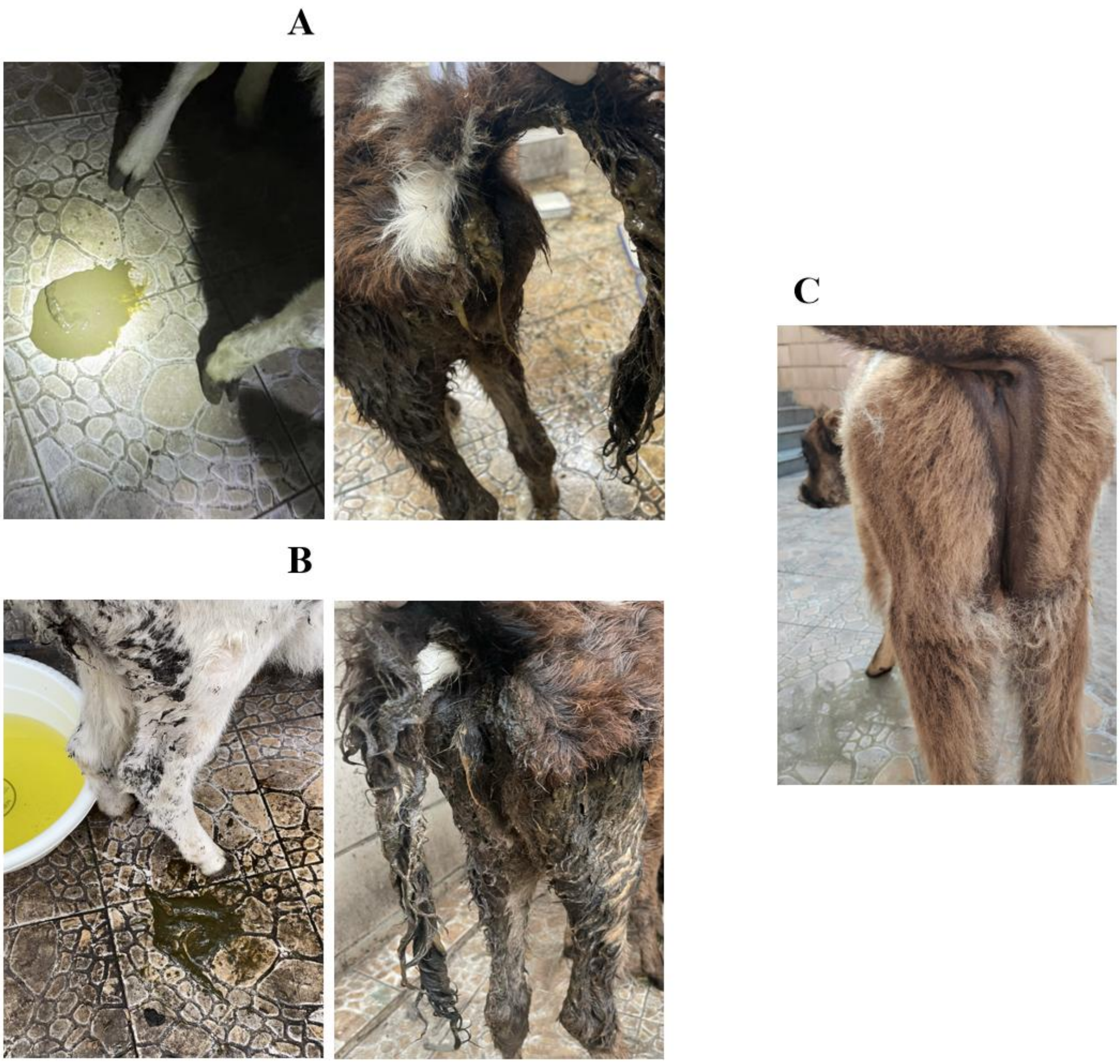

Fig S7. **Yaks in the infection group had severe diarrhea at 3 dpi and 6 dpi.** A: infection group yaks in 3 dpi; B:infection group yaks in 6 dpi; C: yak in control group.

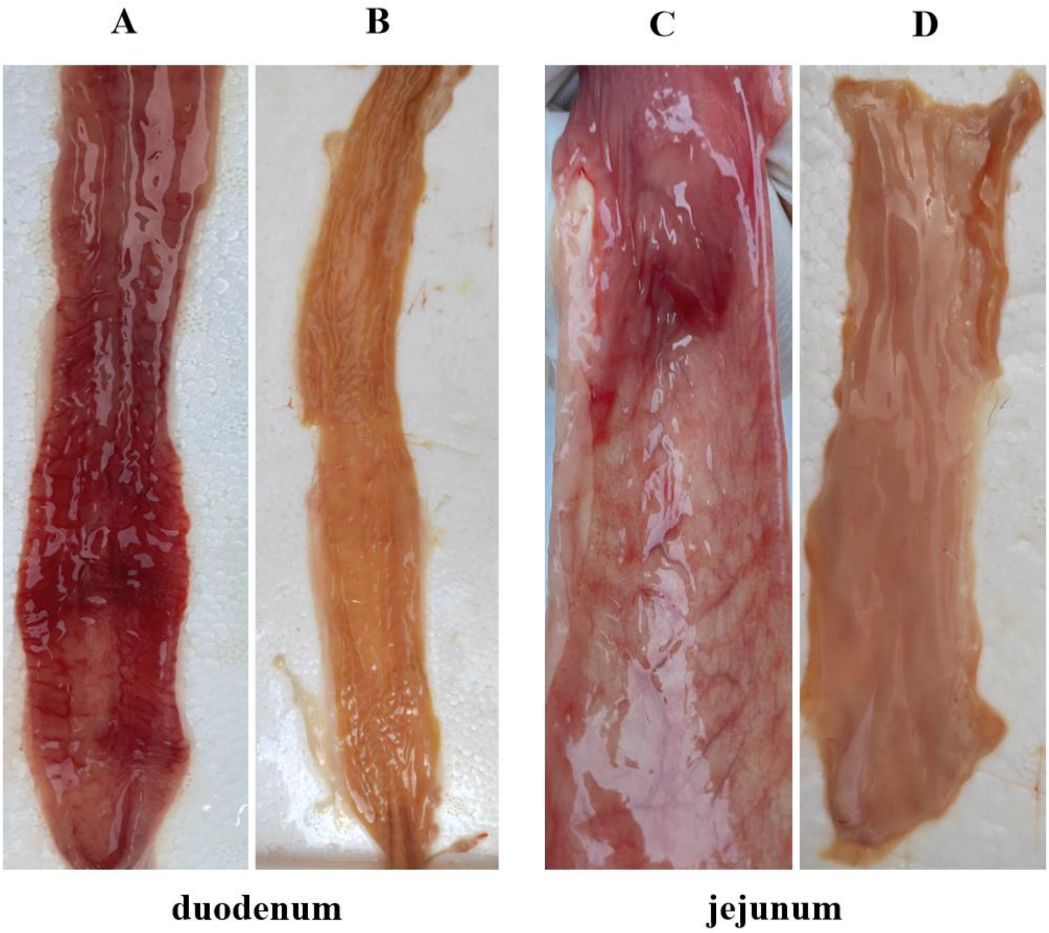

Fig S8. **Pathological changes in the duodenum and jejunum.** (A) & (C) Duodenum and jejunum in the infection group, characterized by intestinal bleeding; (B) & (D):Duodenum and jejunum in controls.

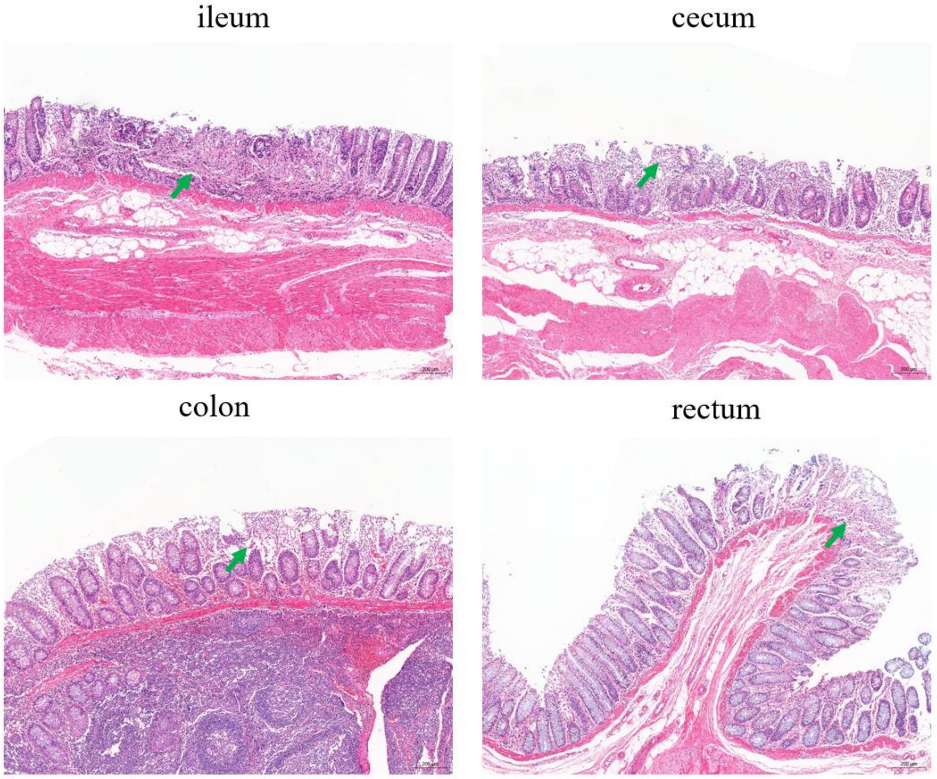

Fig S9. **H&E staining effects in ileum, cecum, colon and rectum after infection with AiV-D (400 X), necrosis of the mucosal layer (↑) and bar, 20 μm.**

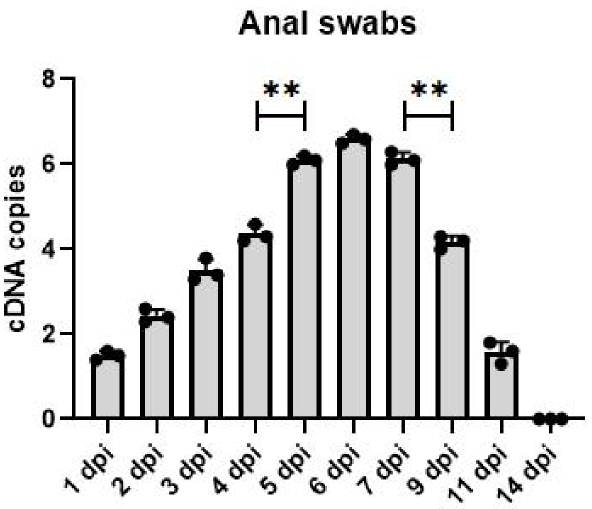

Fig S10. **Dynamic changes on shedding virus of experimental infection of yaks with AiV-D.**
